# Amplifying School Mental Health Literacy Through Neuroscience Education

**DOI:** 10.3390/bs14110996

**Published:** 2024-10-25

**Authors:** Peter J. Vento, Steven B. Harrod, Brittany Patterson, Kristen Figas, Tucker Chandler, Brooke Chehoski, Mark D. Weist

**Affiliations:** 1 Department of Psychology, University of South Carolina, Columbia, SC 29208, USA; harrods@mailbox.sc.edu (S.B.H.); kfigas@email.sc.edu (K.F.); etc3@mailbox.sc.edu (T.C.); chehoski@sc.edu (B.C.); weist@mailbox.sc.edu (M.D.W.); 2 National Center for School Mental Health, University of Maryland School of Medicine, Baltimore, MD 21201, USA; bpatterson@som.umaryland.edu

**Keywords:** mental health literacy, neuroscience education, school mental health

## Abstract

Children and adolescents face a wide variety of developmental changes and environmental challenges, and it is estimated that at least one in five children aged 3–17 will experience behavioral or mental health issues. This period of life coincides with major changes in brain structure and function that have profound long-term consequences for learning, decision-making (including risk taking), and emotional processing. For example, continued development of the prefrontal cortex in adolescence is a sensitive period during which individuals are particularly susceptible to risky behaviors, environmental stressors, and substance use. While recent advances in mental health literacy programs have paved the way for increased awareness of the benefits of mental health curricula in schools, these efforts could be greatly bolstered with support in basic neuroscience education in developmentally appropriate and area-specific content. Here, we provide a discussion on the basic structural and functional changes occurring in the brain throughout childhood, how this contributes to changes in cognitive function, and the risk factors posed by early life adversity, stress, and drug use. Finally, we provide a perspective on the benefits of integrating findings from the field of neuroscience and suggestions for tools to better equip students, teachers, administrators, and school mental health staff to provide new directions for addressing the mental health crises faced by millions of children and youth each year.

## 1. Introduction

In December 2021, the United States (US) Surgeon General issued an advisory calling for action to address the nation’s youth mental health crisis [1]. Prior to the COVID-19 pandemic, nearly one fifth of children and adolescents met criteria for a diagnosable mental health disorder and more than one third were considered at high risk of developing a disorder in the future [2,3]. These concerns were only intensified by the COVID-19 pandemic, with 55% of parents reporting their children were more “sad, depressed, or unhappy” during it [4]. Despite the growing impact of mental health challenges on children and youth, only half of those in need of mental health services receive them [3,5]. Common barriers to access to mental health care include low perceived need, inaccurate mental health knowledge, and stigma [6,7,8].

Schools are recognized as a key location for youth to receive mental health services and support, with over half of youth who receive mental health care accessing it through schools [9]. The growing scale of student mental health needs, combined with shortages of mental health staff in schools, indicate a critical need for universal approaches that can be delivered by existing school staff to all students to promote mental health and prevent the emergence or escalation of mental health challenges [10]. Mental health literacy programs are a promising approach, as they are designed to be delivered universally, and directly address barriers to mental health care.

## 2. Mental Health Literacy

Mental health literacy (MHL) is defined as “knowledge and beliefs about mental disorders which aid their recognition, management or prevention” [11]. This includes recognizing symptoms of mental health challenges, understanding causes and risk factors, and navigating treatment options [11]. MHL also encompasses understanding how to promote and maintain mental health, as well as prevent mental health challenges [12]. MHL plays a pivotal role in numerous mental health and related outcomes. In particular, mental health stigma is associated with an array of poor psychological (e.g., self-esteem and self-efficacy), interpersonal, and life (e.g., employment and housing) outcomes [13]. Mental health stigma is also associated with increased mental health symptoms and worse perceptions of physical health, as well as ineffective coping styles, lower treatment adherence, poorer attitudes toward treatment, and reduced intention to seek treatment [13]. Conversely, accurate mental health knowledge and positive attitudes toward treatment are associated with enhanced help-seeking, treatment access, and well-being [14,15]. Given its role in nurturing favorable treatment access and mental health outcomes, MHL is a meaningful target for efforts in schools focusing on effective prevention, early intervention, and treatment, as in the increasingly prominent multi-tiered systems of support in schools involving education-mental health system integration [16,17]. Furthermore, MHL has been identified as a contributor to the underutilization of mental health services in traditionally marginalized communities and a means of rectifying long-standing treatment disparities adversely affecting Black, Indigenous, and other people of color [18].

There is a growing body of research examining approaches to improving MHL, investigating programs for adults and youth in a variety of settings (workplaces, K-12 schools, universities, online). School MHL programs include initiatives designed to equip students, educators, and/or school staff with knowledge, attitudes, and skills to identify and understand youth mental health challenges, encourage effective coping skills and help-seeking, and engage in behaviors that promote mental well-being [19]. School MHL programs delivered to students are associated with improvements in adolescents’ mental health knowledge, attitudes toward mental illness (i.e., stigma reduction), and help-seeking behavior [20,21,22,23,24]. Moreover, educators and school staff trained in MHL evidence similar improvements in knowledge and attitudes and are better equipped to recognize early signs of distress in students and provide appropriate support or referrals to mental health services [25,26]. Thus, school MHL programs designed for both students and staff are an effective strategy for developing foundational skills and attitudes about student mental health.

The rationale for further developing and implementing MHL programs follows the premise of the health literacy movement: if individuals understand the root causes and bodily processes involved in their health, have a promotive attitude toward their health, and know how to advocate, communicate, and seek help or other resources, they are more likely to take actions that improve their health. While MHL was developed from the health literacy movement [11,27], these fields have since diverged, with MHL growing separately from physical health disciplines including the field of neuroscience, which focuses largely on the biological underpinnings of mental health, cognition, and behavior. Given the linkages between physical and mental health, and their association with numerous other educational and life outcomes, integrating these fields offers promise for improving the holistic well-being of youth [28]. Indeed, the benefits of integrating training and instructional content on the brain-based explanations of developmental, cognitive, social, and emotional processes is a core tenant of the growing field of Educational Neuroscience [29], and when paired with MHL training, it has been shown to reduce stigma, improve recognition of social and emotional challenges experienced by children, and improve attitudes towards people with mental illness [30]. Thus, this paper builds from these observations to suggest a more robust interface between MHL and neuroscience education, pointing to important directions that we predict will enhance both fields, and create new high-impact avenues of interconnected research, policy, and practice.

## 3. Mental Health Literacy and Neuroscience

The mind and body are often viewed as separate, but mental health and physical health are deeply intertwined. For example, individuals with physical illnesses, such as chronic pain or diabetes, are at increased risk of experiencing comorbid depression when symptoms are not well-managed [31]. The mind–body relationship is also present when mental health diagnoses are the primary challenge. Adults diagnosed with mood, anxiety, substance use, and impulse control disorders are more likely to experience a myriad of chronic physical health conditions, including arthritis, heart disease, stroke, hypertension, diabetes, asthma, chronic lung disease, and ulcers [32]. Similarly, adolescents with mood and disruptive behavior disorders experience higher rates of infectious disease, respiratory problems, and weight problems [33]. Individuals with depression are more likely to experience dietary issues and physical ailments than non-depressed counterparts due to related symptoms [32]. Schizophrenia, a severe mental illness, is also associated with chronic physical ailments and shorter life expectancy [34,35]. Co-existing mental and physical health conditions bidirectionally exacerbate symptoms and can complicate treatment plans when interrelated challenges are addressed in silos. Thus, acknowledgment and an integration of the mind–body connection are crucial for efforts dedicated to advancing mental health and well-being in general populations and for children and youth in schools.

MHL offers a vehicle for integrating the mind–body connection into the promotion of well-being in children and communities. Further, neuroscience has informed the development of social–emotional learning (SEL) curricula, and neuroscience content has been successfully integrated into teacher training on the delivery of social–emotional learning curricula, such as the Promoting Alternative Thinking Strategies (PATHS) program [36]. Providing teachers with information on basic concepts in developmental neuroscience builds the rationale for these programs, promoting buy-in and effective implementation. Infusing neuroscience into school MHL programs has a high potential to offer similar benefits for teachers, as well as students. For instance, improving teachers’ understanding of the brain-based underpinnings of mental health can help them understand their students in a new way (and show increased empathy and compassion) and buy into mental health promotion and intervention initiatives [30]. Similarly, sharing foundational neuroscience material with youth in a developmentally appropriate way may help students achieve greater understanding of their own mental health and improve learning outcomes [37]. Results from a recent intensive online search using Google and Microsoft search engines, however, found only a limited number of readily available MHL online resources that, to varying degrees, included at least some reference to the biological bases of mental health and the developing brain (including, but not limited to, “School Mental Health Ontario” https://smho-smso.ca (accessed on 1 August 2024); “Know Before You Go” and “Teach and Learn- Mental Health Literacy” https://mentalhealthliteracy.org/ (accessed on 1 August 2024); “Pathways To Empower” https://www.pathwaystoempower.com/ (accessed on 1 August 2024)). Resources such as these present a tremendous opportunity for improving accessibility and implementation of neuroscience-related content, but more work is needed to incorporate such approaches with broader MHL efforts and evidence-based practices across the globe. Accordingly, the following sections provide an overview of brain development and major findings relevant to youth mental health. We then identify opportunities for integrating neuroscience into school MHL and expound on strategies for bringing neuroscience into the classroom, concluding with a discussion on implications for the broader field of school mental health and priorities for research and practice.

## 4. Brain Development Across Childhood and Adolescence

The brain is the most sophisticated organ in the human body. The brain’s basic structure is highly heritable; however, environmental experiences greatly influence its architecture and contribute to its overall functioning throughout its lifespan. Many assume that critical brain development processes are complete by school-aged years; however, it is now known that several key structures and functions of the brain continue developing well into early adulthood. The brain’s ability to change over time, also referred to as plasticity, enables this continued growth, and the brain has increased capacity for change (is more plastic) during specific periods of life. These sensitive periods for structural and functional development are particularly notable throughout childhood and adolescence where robust periods of growth and refinement occur. During these sensitive periods in development, the brain is especially susceptible to perturbations from environmental factors that can have a profound and long-lasting influence on brain function. Therefore, youth-serving professionals should be aware of brain development basics to help promote social, emotional, behavioral, and academic health and success among children and youth through early, timely, and effective intervention planning.

### 4.1. Early Childhood (Birth to 3)

Early childhood is a critical period for healthy brain development because quantitative and qualitative growth far exceeds changes observed throughout the lifespan [38]. Brain volume doubles in the first year of life and reaches 80% of its adult volume by the age of 3. The brain of a two or three year old child has twice the number of synapses, or the sites where communication occurs between cells, compared to the adult brain. Synaptic connections are formed at their highest rate during early post-natal years, and this rapid increase in connectivity paves the way for later refinement and tuning of brain circuits, which is heavily influenced by environmental factors. Indeed, this developmental window represents a sensitive period as experiences, whether positive or negative, shape brain architecture and create the foundation of potential for future, higher-level cognition.

A notable example of the rapid changes in early-life brain development is the cerebellum, which triples its prenatal size during this first year of infancy and contributes to the development of essential motor skills (e.g., rolling, crawling, walking). Visual areas of the cortex also experience significant growth as the vision of infants improves from limited sight to binocular vision. Paired with the development of the hippocampus (associated with memory) and limbic system (associated with emotion processing, reward, and motivation), infants’ abilities to recognize and distinguish faces, emotions, and intention is drastically enhanced within the first few months of life. Frontal and temporal language circuits also undergo critical changes in the first six months, and their architecture is strongly influenced by languages spoken within an infant’s environment. For example, the brains of infants are equipped for exposure to every language and can distinguish between sounds spoken in native and other languages from birth to 6 months of age. With ongoing exposure to the language spoken in their home, infants’ sensitivity to other languages decreases, and the ability to detect sounds of foreign languages is lost by the age of one. This loss of sensitivity, or perceptual bias, promotes specialization in the native language, which is crucial for early language development and associated with future linguistic abilities. Specifically, better native language sound detection in babies predicts better language skills in the future [39]. As described by Tierney and Nelson [38], “the foundations of sensory and perceptual systems that are critical to language, social behavior, and emotion are formed in the early years”, and the quality of these experiences drives subsequent development.

Early childhood experiences can impact brain architecture in a more substantial manner than those that occur later in life. For example, the Bucharest Early Intervention Project (BEIP) demonstrated the sensitivity of young children to the conditions of institutionalization and the severity of consequences for brain architecture and behavior [38,40]. Patterns of stunted or delayed physical and cognitive growth were observed in populations deprived of healthy experiences while institutionalized. The timing of experiences also matters, as children institutionalized after age two presented with more pronounced social, emotional, and behavioral challenges than those removed from such settings before the age of two. Similar trends have been observed for language and IQ. Beyond deprivation of key sensory and perceptual input (e.g., playing, laughter, language), exposure to prolonged or repeated stress (e.g., physical abuse, adverse childhood experiences) damages the developing brain and disrupts limbic system, frontal lobe, and hippocampal functioning, which is proportional to the amount of stress exposure. The more chronic and severe the early-life adversity is that a child experiences, the more likely they are to experience poor learning outcomes, maladaptive behavior, and mental health challenges in the long term. Taken together, it is crucial that adverse circumstances are remediated as early as possible before the potential of neural development has crystalized and the guardrails on cognitive function have been set.

### 4.2. Adolescence (13–18)

In addition to the substantial proliferation in the number and connectivity of cells in a developing brain through early childhood, the brain undergoes another profound period of maturation through teenage adolescence. The adolescent brain leverages neurological foundations established during childhood to advance physical, cognitive, emotional, and perceptual capabilities. The brain also continues to undergo critical restructuring through this period leading to persistent psychological and physiological vulnerabilities during this timeframe. Structurally, total brain volume decreases in adolescence through early adulthood largely through the process of maturation and refinement of the cerebral cortex. This period is most notably marked by synaptic pruning whereby inefficient neural pathways are degraded and removed, while healthy connections in the brain are strengthened and maintained. Together, these volumetric changes help to facilitate increased connectivity, proper allocation of cognitive resources, and efficient communication across diverse brain regions.

In early adolescence, subcortical structures affecting emotion regulation, motivation, reward, and cognition undergo substantial changes as well. Studies of adolescents indicate changes in connectivity of the nucleus accumbens, putamen, and caudate (part of the basal ganglia), as well as the amygdala, which, together, contribute to refinement in reward learning and emotional processing [41]. Maturation of this system results in heightened sensitivity to rewards in adolescents, while increased activity in these regions helps to contextualize risk-taking behavior commonly observed in adolescence, such as unprotected sex and substance use.

## 5. Environmental Effects and Risk Factors on Brain Development, Cognition, and Behavior

Through a general understanding of the basic neurodevelopmental milestones and the trajectory of typical brain development in early life, a clearer picture of the risk factors affecting children and adolescents begins to emerge. In the same way that basic MHL can help to identify and allocate resources and reduce stigma associated with mental health, an improved understanding of the biological roots underlying mood, decision-making, and learning can help individuals and communities identify and reduce risks to maladaptive neurodevelopment such as chronic stress, community violence, abuse and trauma, and exposure to drugs of abuse. For example, to the developing brain, environmental factors including lack of sleep, poor nutrition, and traumatic experiences are all seen physiologically as stressors that engage compensatory mechanisms to inhibit the growth of new brain cells (neurogenesis), impair healthy connectivity and communication between distributed brain regions, and suppress immune system activity critical for fighting illness and disease.

Many of the brain regions described above that undergo rapid changes during this period of childhood and adolescence, including the prefrontal cortex and hippocampus, are particularly susceptible to these environmental stressors, leading to impaired decision-making, poor planning, and difficulties in learning and attention. While the brain is an incredibly powerful and resilient organ, the changes that occur during these sensitive periods of neurodevelopment are critically regulated by an interaction between our genetic make-up and the environments to which we are exposed, thereby marking the upper and lower boundaries of potential for brain function. The consequences of impaired growth and connectivity during this phase of life are, therefore, often irreversible once individuals reach young adulthood. In turn, promoting healthy lifestyles in school-aged children and youth and increasing awareness of MHL to direct mental health resources to individuals, schools, and communities can have long-lasting consequences in academic/job performance and future social and emotional well-being. Conversely, decreased awareness of MHL and lack of access to mental health and physical health resources can set the stage for persistent elevated risk that extends through life.

Adolescents possess intricate and complex decision-making abilities [42]. Extensive research has focused on the neurobehavioral aspects of risk-taking behavior in adolescence, particularly the neurodevelopmental changes that make adolescents prone to behavioral issues and adverse outcomes [43,44]. While the developmental and clinical sciences acknowledge the potential harm from risky behaviors, recent neuroscience theories of adolescent decision-making present risk-taking behavior as a prosocial adaptation. This perspective helps educators not solely view it as a deficit in neurodevelopment that leads the young person down a dangerous, primrose path but as a behavior that can lead to greater independence and exposure to novel stimuli and interactions [42,45,46].

The mesocorticolimbic dopamine system (MCDS) is an integral neural network for learning and decision-making that regulates goal-directed behavior [47,48]. In the mature adult brain, structures within this system collaborate to balance the pursuit of immediate and long-term goals that include reward seeking and inhibitory control (e.g., self-control), which requires coordination with the fully maturated prefrontal cortex. During adolescence, the MCDS undergoes a substantial reorganization, with the prefrontal cortex (PFC) and deep brain structures such as the nucleus accumbens (NAc, critical for reward learning) experiencing increased levels of neurochemical communication, particularly the chemical messenger dopamine [43,49]. These neural changes are believed to drive the individual towards new learning contingencies in adolescence, which is a crucial element in understanding adolescent risk-taking behavior.

The dual-systems theories of adolescent risk-taking behavior propose that the MCDS plays a crucial role. These theories suggest that the PFC and NAc mature at different rates, leaving one relatively immature. This imbalance in maturation is the prosocial adaptation that characterizes adolescent decision-making. Specifically, the dopaminergic tone rapidly increases in the NAc in adolescence, rendering animals increasingly motivated to engage with novel stimuli and other individuals. Likewise, the dopaminergic tone and dopamine-sensing receptors in the PFC also increase, allowing the frontal cortex to coordinate activity with the MCDS for appropriate decision-making. During adolescence, maturational imbalance occurs between relatively immature inhibitory systems in the PFC responsible for self-control and the enhanced functioning dopaminergic (pro-reward seeking) signaling in the NAc [42,45,46]. Thus, the imbalance drives behavior that is highly attuned to peers and other relevant environmental stimuli. The young person’s executive functioning capacity remains immature, for example, in its ability to fully apply self-control and “just say no” to events and stimuli influenced by the heightened dopaminergic signaling in the NAc.

Given the recent attention that dopamine has garnered in popular culture and social media, it is tempting to interpret neurodevelopmental alterations in the MCDS as increasing “pleasure” or euphoria through increased dopaminergic tone. This line of reasoning (i.e., increased dopamine equals increased euphoria) follows earlier, but since updated, hypotheses of dopamine function, which stated that this molecule produces pleasure or reward in response to consummatory behavior [50,51]. Although beyond the scope of the present review, more recent findings contradict such assumptions and do not support the idea that dopamine makes humans *feel* rewarded. Briefly, dopamine is released in response to reward and punishment, indicating that this neurotransmitter signals the experience of ecologically relevant stimuli that produce favorable and adverse outcomes, respectively [52]. Furthermore, several studies suggest that dopamine release represents “craving or wanting”. Dopamine is released in the MCDS when the individual perceives a cue previously associated with a rewarding stimulus through Pavlovian conditioning [53]. Thus, dopamine is released to signal the occurrence of conditional stimuli learned about in previous environmental contingencies and is often discussed as supporting “anticipation” of an upcoming event. Finally, dopamine likely also plays a role in effort associated with motivational outcomes. Drugs that block dopamine receptors also decrease the initiation of motoric behavior involved in approaching a goal box containing rewarding food [50,54]. Together, these findings indicate dopamine is not the pleasure neurotransmitter, per se; instead, it signals relevant experiences in the environment and subsequently “anticipates” the occurrence of biologically relevant events.

Interestingly, the MCDS undergoes a final developmental alteration as the brain matures. As the individual approaches young adulthood, the enhanced dopaminergic tone and communication throughout the MCDS revert to preadolescent levels and do not return [49]. Given that risk-taking behaviors recede during adulthood, the dual-systems approach reasonably explains the vacillations in risky adolescent choice behavior [42,45,46].

Why should this neuroscience-related content interest the community of teachers, school administrators, school mental health staff, and parents? Clearly, increases in wanting, craving, and anticipation of interactions with peers and other high-value stimuli, such as sex, drugs, social media, and video games, are noticed and documented in various ways by parents and teachers. Undoubtedly, the adverse outcomes of risky behaviors pose multiple challenges to family, law enforcement, schools, and public health professionals [42]. Interpretations of risk-taking behaviors that rely on notions of parental authority (e.g., *why do you behave that way when I told you not to?*), learning deficits, memory loss (e.g., *did you not learn your lesson the first time?*), morality (e.g., *good people do not have sex outside of a committed relationship*), and shame (e.g., *I do not know who you are*) are unreasonable strategies if we consider the source of such craving, according to neuroscience models of adolescent choice behavior. The dual-systems model of adolescent risk-taking offers more than theoretical insights by shedding light on the complex interplay between neurodevelopment and risky choice behavior. It provides educators a unique window to understand and address adolescent risk-taking behavior compassionately and without stigma, fostering a more supportive learning environment.

## 6. Integrating Neuroscience into School Mental Health Practices

There are opportunities to integrate neuroscience and MHL through various school structures and practices. As mentioned, multi-tiered systems of support (MTSS) offer a framework with which to conceptualize and organize these initiatives. MTSS articulate key systems practices (e.g., screening, teaming, data-based decision-making) and define a continuum of support to ensure all students have access to resources and interventions matched to their needs. This continuum includes Tier 1 (i.e., universal) support that is available to all students and staff, typically delivered to an entire classroom, school, or district. Tier 2 (i.e., targeted) programs are delivered to a subset of students who display a need for greater support. Tier 3 (i.e., intensive) interventions are comprehensive, individualized interventions delivered to a small number of students with the most intensive concerns. Ideally, these MTSS programs are developed collaboratively by leaders pursuing true education–mental health systems integration, with community-based clinicians augmenting the work of schools, adding value to both systems (e.g., increased resources and support to schools, increased relevance and accessibility of the mental health system [16]).

## 7. Integration at Tier 1

There is a rich opportunity at the intersection of neuroscience and MHL for school staff to build impactful relationships that contribute to an overall positive school climate and positive outcomes for students. Bessel van der Kolk, author of The Body Keeps the Score [55], explains that we are close to becoming a trauma-informed society, and there are critical choices to be made to keep pace with the ever-growing mental health needs of youth. This charge to foster safety and belonging is of utmost importance to our efforts to better support the mental health needs of children. Developing meaningful relationships at schools is an important protective factor for youth. These meaningful relationships, described by the term “school connectedness”, are defined by a sense of belonging, being supported, and feeling cared for at school [56]. Students who feel connected to their school are less likely to experience negative mental health outcomes, sexual health risks, substance use disorders, and violent crimes, and are more likely to experience positive academic outcomes [56,57].

A variety of universal strategies can be applied school- and classroom-wide to increase awareness for and the application of “neuroscience integrated mental health literacy” (N-MHL). For example, MTSS teams can leverage school–community partnerships to disseminate useful information about N-MHL to students, parents/caregivers, and all school-based staff (e.g., educators, related mental health professionals, support staff). Consider the use of well-established in-person events (e.g., Back to School Night, Math Night, sporting events) and electronic platforms (e.g., Google Classroom) to maximize the reach and accessibility of N-MHL content.

High-quality professional development opportunities may further promote the importance of N-MHL and generate educators’ interest in implementing classroom-wide programming. Introductory training should address the what, why, and how of N-MHL. Specifically, professional development objectives may aim to (1) define N-MHL, (2) illustrate the importance and impact of N-MHL for students and teachers, and (3) clearly articulate the role of educators and other school-based staff. Addressing the role of educators further includes provision of relevant intervention resources, implementation assistance, and ongoing training or consultation to promote fidelity.

Within the classroom, N-MHL can take many forms. Perhaps the most straightforward approach is for educators, school counselors, or psychologists, to deliver lessons focused on neuroscience and MHL, using and/or augmenting existing MHL curricula like the Guide, one of the original and most widely implemented MHL programs originally designed for high school students [23,25,58]. The Guide is an evidence-based, classroom MHL curriculum consisting of modules focused on stigma and reducing it, understanding mental health, mental health challenges, and seeking help/finding support, with positive outcomes of increased mental health knowledge and reduced stigma for participating students. The Guide program was originally developed in Canada as a framework to implement MHL content in schools and is now implemented in numerous other countries, including the United States, the UK, Malawi, Nicaragua, Tanzania, and Wales. By providing training opportunities and freely accessible lesson materials such as this, focused on N-MHL, educators expand their capacity to reach and connect with students on important topics they might not otherwise discuss. For example, by discussing brain development and its impact on mental health during adolescence, teachers provide students with important scientific context related to development and can discuss challenges that might arise when seeking to maintain mental wellness. As appropriate, teachers are encouraged to share accounts of their own experiences with mental health, and by doing so, model vulnerability and openness to their students who can, in turn, share their struggles with a trusted adult or peer at school. By opening up about their mental health, teachers not only create in-roads by which students can connect to support, but also provide the important opportunity for students to see them as fellow humans who also experience challenges with mental health, rather than as “just a teacher”. Classroom conversations about brain development and mental health help to create, foster, and maintain safe, supportive environments where students can explore their emotions, learn basic coping skills and strategies for maintaining mental wellness, or alert a teacher about a mental health challenge that may require more intensive services. N-MHL lessons provide a structure for having these conversations in a way that may feel familiar to teachers and students.

While a teacher-delivered N-MHL curriculum is a strong option, the current reality is that teachers are experiencing high levels of stress and burnout [59], and many do not have the time or energy to investigate the benefit of such practices, much less adopt them in their classrooms. Thus, we must provide strong, clear connections so teachers see the relevance and necessity of integrating such curricula into their usual classroom lessons. Additionally, creating realistic and sustainable opportunities to deliver such curriculum is vital. Pre-packaged, ready-to-use curricula, lessons, and activities, such as the Guide, Teach Mental Health Literacy (https://pdce.educ.ubc.ca/teach-mental-health-literacy/, accessed on 1 August 2024), Classroom WISE (https://www.classroomwise.org/, accessed on 1 August 2024), and the recently developed brain-based MHL content “Pathways to Empower”, are freely available online (https://k12.kendallhunt.com/program/pathways-empower, accessed on 1 August 2024). Required training should also occur during traditional work hours and be integrated into existing professional development procedures. Additionally, partnerships with regional external experts in neuroscience and mental health may be considered to deliver this information to students as part of existing initiatives in neuroscience outreach from organizations, such as the Society for Neuroscience annual Brain Awareness Week program (BrainFacts.org). Indeed, many neuroscience departments and graduate training programs in biomedical science often have programs specifically designed to support outreach in neuroscience education, but leaders often do not know how to direct these resources where they are needed the most. There is certainly much room for growth in building partnerships with such experts, as many of these programs are currently held in complete isolation from one another, but considerations should be made to include teachers or mental health counselors within schools as active members of such a partnership so that students have a familiar, trusted adult with whom they are already connected.

A complementary approach is to integrate N-MHL content into daily classroom rhythms and existing curricula. Infusing morsels of N-MHL content in daily check-ins and discussions can be a path to fostering strong, safe relationships with students. Rather than creating “one more thing” for teachers and school-based staff to implement, developing meaningful relationships with students should be treated as an ongoing, foundational practice to be supported and sustained through additional school counselors and staff, rather than a time-limited initiative with an endpoint. N-MHL can also be integrated into most traditional K-12 courses, such as science, health education, language arts, and social studies. In science, brain curriculum can be augmented to include discussions about primary functions and how they impact student feelings and behaviors. Health education courses can naturally integrate N-MHL by naming sections of the brain associated with physical health changes during puberty. Language arts educators may integrate questions about feeling–behavior connections into reading-based discussions. N-MHL can also be incorporated into social studies classes through history reports on mental health pioneers or research on experiences influencing student mental health. Finally, given the connections between physical and mental health, N-MHL may be a natural fit for health and physical education courses, expanding students’ knowledge about how to promote and protect their holistic well-being.

## 8. Integration at Tiers 2 and 3

Neuroscience can also be integrated into MHL in the context of intervention for students requiring more intensive forms of support. At Tiers 2 and 3, this integration becomes more specific to the particular challenge(s) confronted by an individual or group of students. While all students may receive basic information on brain development and functions, as well as structures and processes linked to mental health, students receiving small-group or individualized intervention may also benefit from neuroscience content that enhances their understanding of their distress and informs the intervention they are receiving. At this level, MHL becomes synonymous with psychoeducation (a term we prefer to avoid) and is most likely to be provided by a school’s mental health professional to students and their caregivers. Others have recommended infusing more neuroscientific content into psychoeducation, identifying the major topics of interest and offering a structure for incorporating neuroscience material in clinical practice [60]. Still, little empirical research has been conducted on this topic. The small body of research that exists suggests that neuroscience-informed psychoeducation may help improve insight into metacognitive weaknesses for individuals with substance use disorders (Rezapour et al., 2021) and help shift attributions of the cause of depression [61]. However, these studies are preliminary and characterized by low dosage (e.g., only one 30 min lecture on neurobiology of depression; [61]) and small sample sizes [62], highlighting the need for additional investigations of the utility and effectiveness of infusing neuroscience into mental health interventions.

Clinicians charged with delivering neuroscience-infused psychoeducation in the context of individual or group counseling may experience similar barriers to those expressed by teachers implementing MHL curricula. Lack of requisite knowledge and familiarity with this approach to psychoeducation may increase clinician discomfort, hindering delivery. Thus, implementing this approach necessitates educating clinicians about neuroscience, training them to provide effective, relevant, and developmentally appropriate neuroscience-infused psychoeducation, equipping them with tools and resources, and ensuring they have access to clinical supervisors who are also versed in this approach. Training clinicians on neuroscience content may be particularly impactful, as clinicians who primarily provide Tiers 2 and 3 mental health services in schools may also consult on and/or help deliver Tier 1 support to students.

School teams provide another opportunity to integrate neuroscience and MHL at the advanced tiers. While formal MHL curricula delivered to students and/or staff are valuable, MHL can be developed through less formal means, such as team discussions. When students are identified as needing support through screening or referral, it is common for a team of school professionals to collaboratively discuss the presenting concern and decide upon an appropriate course of action. These initial meetings, which may include caregivers, are an opportunity to provide mental health education through N-MHL to caregivers and/or other team members to build their understanding of the child and the factors contributing to their current challenges. These conversations need not be limited to initial meetings and may also occur at review meetings, such as for students who are currently on an individualized education program (IEP). As members of the team (e.g., teachers) shift as students matriculate through the grades, there are new opportunities to disseminate relevant neuroscience material and continually develop N-MHL content.

## 9. Conclusions and Considerations for Effective Integration

Developing partnerships between education, mental health, and neuroscience is necessary to ensure neuroscientific content is accurately distilled, translated, packaged, and delivered to the appropriate audience [63]. Partners from each of these disciplines should collaboratively define relevant neuroscience content to be shared with various audiences, such as educators, school mental health staff, students, and families. Neuroscience material should be collaboratively translated to align with the audience’s presumed baseline knowledge and developmental level and be clearly linked to the purpose of sharing the information. Cross-disciplinary partnerships can also be leveraged to develop engaging and effective activities and develop new and innovative methods for delivering content that is feasible, acceptable, and fits with the school context.

Just as abundant as the opportunities to integrate neuroscience and MHL, so too are the challenges to engaging in this work. Even when some MHL curricula (such as the Guide) include content on neuroscience, there are a number of barriers to delivering this content effectively. Indeed, in a 2020 study involving 968 preservice teachers in Hong Kong, it was found that most preservice teachers have limited knowledge of the brain and often hold misconceptions of the role of neurobiology in cognition and behavior [64]. Authors of this paper (MW, BP, BC, TC) are currently leading the first US randomized controlled trial to investigate outcomes associated with the implementation of the Guide within American schools, which includes enhanced content on the brain and neurodevelopment geared towards middle school students. This middle school version of the Guide is freely available at the program website: https://www.partneringforstudentwellness.com/, accessed on 1 August 2024. While this study remains ongoing, we have found similar sentiments in annual focus groups where teachers report mixed feedback on neuroscience content, with some voicing discomfort with the content and a tendency to skip and/or minimize lessons on these topics. Others expressed that the content seemed too detailed and advanced for middle school students. For these reasons, lessons including neuroscience content were among the least preferred by the focus group participants (unpublished finding). These findings suggest that infusing neuroscience into school MHL curricula poses challenges for both teachers and students, indicating a need for an enhanced and more tailored content-specific approach to this integration, perhaps through supplying some basic neuroscience education to teachers while enhancing existing infrastructure and training for school counselors and psychologists [65]. It is, therefore, imperative to assess staff’s comfortability with neuroscience content, as well as their interest in and willingness to implement different N-MHL approaches. Provider competence, motivation, and perceptions of acceptability and feasibility are a few of the key factors to consider when implementing any new initiative [66] and are likely to influence uptake of N-MHL. Similarly, educator and leadership buy-in are critical to implementation success [67]. By supporting and prioritizing the N-MHL initiative, promoting a supportive school culture inclusive of the new initiative, and allocating resources to support implementation, school leaders directly and indirectly influence implementation [68]. Additionally, competing initiatives, such as academic curricula, social-emotional learning (SEL) programs, and a glut of intervention options, can overwhelm efforts to adopt a new practice, requiring streamlining, integration, or concurrent de-implementation of other initiatives to encourage uptake of N-MHL [68,69]. The competition of responsibilities and priorities, including prioritization of academic material (particularly STEM) over arts and supplementals, can result in reduced health education opportunities, stymieing efforts to implement N-MHL.

It follows that effectively implementing N-MHL requires building knowledge and skills, developing buy-in and enthusiasm, and ensuring there are adequate opportunities and resources. Buy-in of N-MHL can be promoted by soliciting educator perspectives and preferences and teaching educators and leaders N-MHL concepts and the rationale behind them. Pruning competing initiatives or streamlining and integrating N-MHL with other initiatives may also enhance buy-in and motivation by reducing burden and increasing feasibility. For instance, N-MHL may be incorporated into existing SEL lessons, providing greater depth and specificity of content on mental health without adding redundancy by delivering an interconnected curriculum. It may also be helpful to highlight the many ways in which N-MHL can be implemented, only a few of which are reviewed in this article. N-MHL does not have to be a big initiative, but it can be integrated into small exercises, such as adding context to deep breathing or mindfulness exercises. This can also be incorporated into professional development or staff meetings. For instance, administrators could start meetings with a quick mindfulness activity, paired with an N-MHL explanation of what occurs in the brain and body during such exercises. This provides an important model, which teachers can then adapt to their classrooms.

Implementing N-MHL in any format requires leaders to define expectations, carve out time and space, and procure needed materials and resources. This may mean allocating time each day, week, or month for teachers or other staff to deliver lessons from a N-MHL curriculum. Or it may mean convening a team to identify opportunities and strategies for integrating N-MHL into existing curricula, which would require developing materials and activities in-house. Notwithstanding, the average educator has limited access to neuroscientists, and neuroscientists may lack the time, incentive, and understanding of schools to instigate these partnerships. This speaks to the foundational need to develop and expand university–school partnerships and develop interdisciplinary communities of practice to support this work. This does not require starting afresh but rather might involve expanding existing partnerships and communities of practice supporting MHL or establishing sub-teams specifically focused on the advancement of N-MHL. There are a range of resources that exist to assist schools in implementing relevant programming, several of which are already noted in the sections above. This includes the Mental Health Literacy Collaborative, a rapidly growing organization in the U.S. whose mission is to “make the education framework of mental health literacy foundational in schools and communities” (see https://www.themhlc.org/, accessed on 1 August 2024). The collaborative works with schools and communities around the U.S. (with developing international connections) aim to assist in MHL advocation and education, connecting to relevant resources and research and providing policy guidance. In addition, relevant resources on the Guide MHL curriculum are available at https://mhlcurriculum.org/ (accessed on 1 August 2024). Through these partnerships, educators, mental health clinicians, researchers, neuroscientists, and/or other healthcare providers can convene to define N-MHL priorities, adapt content to different providers (e.g., teachers, administrators, mental health clinicians), and tailor content and activities to students of various developmental levels.

## Data Availability

No new data were created or analyzed in this study. Data sharing is not applicable to this article.
